# Human Temperaments. III.—The Temperament of the Artist


**Published:** 1916-05-27

**Authors:** 


### HUMAN TEMPERAMENTS.

### III.—The Temperament of the Artist.*

As has already been said, the temperament of the artist is very different from the artistic temperament. The respects in which they differ will appear in the following examination. They are wholly different temperaments, but they are not wholly incompatible. An artist is no more than other men immune from the artistic temperament. He may have a certain infusion of it, and in so far as he is infected with it he is in so far less capable as an artist. The artistic temperament exists in all grades and degrees, and is far from always present in its full development. An artist, like anyone else, may be besmirched with some small degree of it, but many artists, and all the greatest artists, are entirely free from any trace of it. There was no trace of the artistic temperament in Shakespeare, Milton, or Cervantes; in Purcell, Beethoven, Mozart, or Mendelssohn; in Titian, Rembrandt, Reynolds, or Turner; in Fielding, Scott, Jane Austen, or Thackeray; in Michael Angelo, Canova, or Flaxman; but Byron had it, Whistler had it, Charles Reade had it, Savage had it, and Wagner had it in high degree.

### What is It.

There are many definitions of art and of the artist, but there is none that will stand examination. The definition or description of art that is at present most in vogue is that it is "self-expression." If this is so, then ill-temper is art, for ill-temper is certainly self-expression. It is perhaps this definition or description that persuades the man of artistic temperament that he is an artist, and that the more he expresses himself—that is to say, the less he exercises self-control, and the more he yields to the impulse of the moment—the greater artist he is. This description of art as self-expression is not merely erroneous: it is pernicious; and it is necessary to find some more satisfactory formula.

Whatever else art may be, it is certainly creative; and the artist, whatever else he may be, is a creator. He does that which no one else has done, and which has never been done before. But stop! Is not the engraver, who copies another man's pictures, an artist, even though he is a copier? Is not the pianist or the violinist an artist, even though he but reproduces the creation of another mind? Yes and no. The engraver is not an artist if he but slavishly copies the painting. The pianist or the violinist is not an artist if he but mechanically reproduces the work of the composer. But the engraving becomes a work of art if and when the engraver, by the exercise of his creative faculty, adds to the engraving qualities which compensate for the loss of colour, and so makes of the engraving not a mere mechanical copy, but in some respects a new creation. The musician may, it is true, be a mere mechanical instrument to reproduce sounds in certain combinations and in a certain order; but he is no artist unless and until he reproduces these sounds in such a way as to add to them new meaning, and thus to create for us something we have never experienced before, even though the music he plays may be familiar enough. The artist is creative. He has originality. He puts things before us in a new light. The thing that he presents to us may be familiar enough, but he represents it to us in a way that is new. He is a creator. Is it, then, a sufficient definition of the artist to say that he is a man who creates new things? Clearly it is not, for the inventor also creates new things; and yet the artist and the inventor, though they have something, perhaps much, in common, are not the same. The discoverer is also, in a certain sense, a creator; and discovery and invention go hand in hand. Yet we should not call Watt, or Bessemer, or Kelvin an artist; still less should we so denominate Newton or Darwin. Clearly, although the artist is necessarily a creator, yet he is not the only creator. He is a creator in a certain field only. Some creations are artistic, others are not.

Evidently, those creations which we call inventions are creations in the realm of utility. The inventor creates that which is useful; but what do we mean by useful? Do we mean that which increases our comfort, which adds to the amenities of life, and brings new pleasures within our reach? Scarcely, for the artist also does this. By that which is useful we mean that which assists us to attain our ends, that which enables us to attain our ends with less expenditure of effort, or that which brings new ends within our reach; in short, that which opens the way to new pleasures. The pleasure that we gain from an invention is not in the contemplation of the invention, but in the use of it; and this is the difference between invention and art. For the pleasure that we gain from an artistic product is not in the use we make of it, but in the contemplation of it. An invention is a means to further ends: an artistic product is an end in itself.

### The Industry of the Artist.

The artist, whatever the medium that he works in and moulds to his purpose, is one who produces what is pleasing in the mere contemplation; and this pleasure in contemplation is the end that he sets himself in his creative work. Now, pleasure in mere contemplation is a luxury that we cannot afford until more urgent wants are satisfied. It is an occupation for idle hours, an occupation that is an end in itself and leads to nothing further, and hence the development of art keeps pace with the increase of leisure amongst those who are capable of enjoying art. This undeniable association of art with leisure leads those of artistic temperament to suppose that by living a life of leisure they are cultivating art; but this is a very erroneous view. The thorough enjoyment of artistic products demands leisure, it is true. The very busy man, the man whose whole time and energies are

* The previous articles appeared on May 6 and 13.

absorbed in the struggle for existence, has no leisure to read poetry, to hear music, to witness the drama, and so forth, and some leisure there must be before these artistic products can be enjoyed; but no leisure went to the creation of these artistic products. The artist himself must live, and does live, a very laborious life. Whatever his material, whether language, pigments, building materials, sounds, marble, bronze, or his own elocution and gesture, the artist must serve a long and laborious apprenticeship before he can attain that complete and facile mastery over his material which will enable him to produce the effects he desires. It is this capacity for long and laborious industry, the fruit of self-restraint, self-control, and self-denial, that so eminently distinguishes the artist from the man of artistic temperament. Carlyle described genius as an infinite capacity for taking pains. The description is ridiculous. If it were true, every ant and bee would be a genius, for no creature has so great a capacity for taking pains; but it has this justification, that without great precedent labour no work of genius ever was, or can be, produced. Great capacity for taking pains no more constitutes genius than a strong wrist constitutes a good fencer or a pair of legs constitutes a good rider; but it is true that without a strong wrist a man cannot be a good fencer; without legs he cannot be a good rider, and without great capacity for industry he cannot be a great artist; and this it is, in the main, that distinguishes the artist from the man of artistic temperament.

### Further Qualifications.

Granted this great capacity for industry, which the artist must possess, but which is by no means peculiar to the artist, what other qualities go to make up the temperament of the artist? As an artist he is to produce something that pleases by the mere contemplation of it. His industry has given him mastery over his materials, but what further qualities must he have to direct this industry into the desired direction? Whatever gives pleasure by the mere contemplation of it must be something that arouses emotion. To arouse emotion it must express emotion; and to express emotion it must be the expression of emotion felt more or less vividly by the creator while he was creating it. Hence the artist must be capable of experiencing emotions. No doubt everyone is capable of experiencing emotions in circumstances calculated to produce emotions, but in such circumstances the emotions are too forcible to allow us to attend to their expression, and the expression is correspondingly crude. In poignant grief, in extreme terror, in racking anxiety, in glorious joy, we are too much overwhelmed with the emotion to be able to give to it adequate verbal expression. To do this we must stand aloof and feel not the emotion itself, but a pale yet accurate image of it, which we must imagine to ourselves. It is this power of imagining emotion in all its complexity, but in attenuated intensity, that lies at the root of the temperament of the artist. He must have the dramatic faculty of placing himself in an imaginary situation, and imagining accurately but faintly how a person really in such a situation would feel, and then of expressing those feelings so as to make them intelligible to a spectator, and so as to arouse in him the emotion not that would be felt by the actual actor, but that which is felt by the artist himself. It is from the contemplation of the expression of emotion, and from experiencing the emotions of a spectator of the actual scene, that the pleasure of witnessing artistic products is derived.

The merit of an artistic product depends on two factors; first, the depth or elevation of the emotion expressed; and, second, the skill with which it is expressed. Shallow and trivial emotion makes but trifling art, however perfect the technique with which it is expressed. Noble emotion of great profundity, expressed crudely and inadequately for want of mastery of technique, is not great art. It is an unsuccessful attempt at great art. The greatest art is that in which the noblest emotion is expressed with the most perfect technique.

### Ars Longa, Vita Brevis.

To attain perfect technique in any art, laborious industry is necessary; but laborious industry alone is not enough. There must be in addition innate aptitude, aptitude that varies, of course, with the medium chosen for the expression. Without this innate aptitude no industry will give mastery over technique: without industrious study and practice no aptitude is of much avail. The combination will give exquisite technique, but technique alone will never make a man an artist. In addition, he must have the capacity of realising and picturing emotion. He must be capable of experiencing the emotion that he is to represent. Many of these are not expressible in words; they have no intellectual equivalent; they can only be felt, and, if expressed, must be expressed inarticulately; and these are the emotions that find expression especially in music and in some forms of architecture; but some of them can also be expressed in gesture, demeanour, and facial alteration; and therefore in the depicting of these on canvas and in the solid the more profound and stirring the emotion the higher the art.

One more quality must be added in order to complete the temperament of the artist. This is the ability to construct an harmonious and consistent plan. Whatever the medium in which the artist expresses himself, whether he is a poet, a dramatist, a novelist, a musician, an architect, a painter, a sculptor, an actor, or what not, each work of art that he produces must be modelled upon a consistent plan. It must have one leading and dominant feature to which all the rest are subordinate and subsidiary in various degrees, with which none must be discordant, and to which none must be irrelevant. The simpler the plan, the more harmoniously its parts are interrelated, and the more variously the different parts illustrate and corroborate the central theme, the greater the work of art.

